# Orexin and cannabinoid systems modulate long-term potentiation of the hippocampus CA1 area in anesthetized rats

**DOI:** 10.22038/IJBMS.2023.73979.16075

**Published:** 2024

**Authors:** Reza Fartootzadeh, Ramezan Ali Taheri, Mohammad Reza Nourani, Kamelya Goudarzi

**Affiliations:** 1 Department of Physiology, Ramsar Campus, Mazandaran University of Medical Sciences, Ramsar, Iran; 2 Department of Physiology, Faculty of Medicine, Mazandaran University of Medical Sciences, Sari, Iran; 3 Nanobiotechnology Research Center, Baqiyatallah University of Medical Sciences, Tehran, Iran; 4 Department of Psychiatry, Ramsar Campus, Mazandaran University of Medical Sciences, Ramsar, Iran

**Keywords:** AM251, Cannabinoid receptors, Hippocampus, Long-term potentiation, Orexin receptors, TCS OX2 29

## Abstract

**Objective(s):**

Long-term potentiation (LTP) is a kind of synaptic plasticity and has a key role in learning and memory. Endocannabinoids and orexins are the endogenous systems that can modulate synaptic plasticity. Given that new studies have shown an interaction between cannabinoid and orexin systems in the brain, we decided to examine this interaction between the two systems on LTP induction in rat’s hippocampus.

**Materials and Methods:**

Twenty-eight male Wistar rats were used for evaluating the effects of co-administrating of cannabinoid-1 receptor (CB1R) antagonist (AM251) and orexin-2 receptor (OX2R) antagonist (TCS OX2 29) on the induction of LTP in the Schaffer collateral-CA1 synapses of rat hippocampus. The drugs were microinjected into the CA1 area of rat hippocampus 30 min before inducing of LTP.

**Results:**

Results showed that sole administration of the antagonists inhibited LTP, with respect to the control group. Also, co-administrating of them reduced LTP as compared to the control group, but not significantly more than that when the antagonists were solely microinjected into the CA1. Nonetheless, the inhibitory effect of concurrent administration of the antagonists on LTP lasted until the end of the recording.

**Conclusion:**

These results propose that endogenous cannabinoids and orexins play a role in the expression of LTP, at least by CA1-CB1Rs and CA1-OX2Rs, respectively. Finally, there is no interaction between CB1R and OX2R on the induction of LTP in the Schaffer collateral-CA1 synapses; therefore, these two systems possibly act through common signaling pathways in the hippocampus’s CA1 region.

## Introduction

It is well recognized that the cellular basis of learning and memory is long-term potentiation (LTP) (1, 2). LTP is a model of synaptic plasticity that is expressed artificially and occurs in some portions of the brain, including hippocampal formation (3). It is generally accepted that LTP happens at synaptic sites during the formation of learning and memory; therefore, synaptic transmissions in neural circuits are not fixed but change under distinct conditions, such as memory formation (3). 

Endocannabinoids, the endogenous ligands of the cannabinoid system, are fatty acid derivatives that affect various physiological functions, like synaptic plasticity, by inducing CB1R activity (4, 5). In reviewing the literature, controversial results have been reported concerning the cannabinoids’ effects on synaptic plasticity. For example, it has been shown that the administration of CB1R agonists exerts inhibitory effects on the induction of LTP (6, 7). On the contrary, the CB1R antagonist impaired LTP induction in the CA1 neurons (8). 

Orexins (orexin A and B) act through two types of G-protein coupled receptors called the orexin-1 receptor (OX1R) and orexin-2 receptor (OX2R) (9). Both orexin receptors are distributed in the hippocampus (10). The orexinergic neurons of the lateral hypothalamus send their projections throughout the CNS and spinal cord, including the hippocampal formation (9, 11). This diffuse projection allows them to regulate various brain functions, including sleep/wakefulness, autonomic function, metabolism, feeding behaviors, addiction, and learning and memory (11-15). A large body of data indicates the role of orexin receptors in learning and memory. For example, Akbari and colleagues demonstrated that OX1R blockade in CA1 and dentate gyrus (DG) impairs spatial learning and memory (16), and also suggested the DG-OX1R involvement in the expression of LTP (17). Moreover, selective blockade of the OX1R impairs consolidation and retention in the passive avoidance learning task, and OX2R inactivation cause a deficit in retention (18). These studies strongly show the involvement of the orexin system in the process of learning and memory. Some studies are showing a vast number of ways to improve memory (19-21).

Finally, anatomical findings have presented that OXRs and CB1R display an overlapping distribution in different regions of the brain (10, 22), supporting their common role in the regulation of some physiological functions, such as reward, appetite, sleep/wake cycle, and nociception, as reviewed by Berrendero *et al*. (23). Moreover, both OX1R and OX2R can create homo- and heteromeric complexes with each other and with the CB1R (24). Besides, an interplay between the cannabinoid and orexinergic systems has been shown within the ventral tegmental area (VTA) and the nucleus accumbens (NAc) (15, 25-27). Despite the evidence about the possible crosstalk between orexin and cannabinoid systems, no study has explored this interaction between CB1 and OX2 receptors in learning and memory regulation. Therefore, here we have considered (i) involvement of OX2R, (ii) involvement of CB1R within the CA1 in the mediating of LTP in rats, and (iii) presence of an interplay between these receptors in this phenomenon.

## Materials and Methods

***Animals***

The tests were executed on the adult male Wistar rats, weighing 230–260 g at the time of surgery. The subjects were kept four per cage with free access to lab chow and tap water under a 12/12 hr light/dark cycle. After at least one week of adaptation, animals were separated into DMSO, TCS OX2 29, AM251, and TCS OX2 29 + AM251 groups. Each experimental group contained seven animals (total number: 28), and each rat was used once. The study was approved by the Ethics Committee of Animal Use of the Baqiyatallah University of Medical Sciences, and all tests were performed in accordance with the National Institute of Health Guide for the Care and Use of Laboratory Animals (NIH Publications No. 80-23), revised 2011.

***Drugs***

The following compounds were tested: urethane (Sigma-Aldrich, Germany), TCS OX2 29 (Tocris Bio-science, Bristol, UK), and AM251 (N-(piperidine-1-yl)-5-(4-iodophenyl)-1-(2, 4-dichlorophenyl)-4-methyl-1H-Pyrazole-3 carboxamide) (Sigma-Aldrich, USA). AM251 (25 ng/rat), as a CB1 receptor antagonist, and TCS OX2 29 (3 ng/rat), as an OX2 receptor antagonist, were dissolved in dimethyl sulfoxide (DMSO; up to 10%, v/v), in a volume of 0.5 μl, and microinjected into the CA1, 30 min before HFS (high-frequency stimulation). The control group received the same volume of DMSO, as the vehicle, by the same route. Concentrations of the antagonists were based on previous studies (27, 28).

***Surgical procedures***

Animals were deeply anesthetized with intraperitoneal (IP) injections of urethane (1.4 g/kg; Sigma-Aldrich, Germany), gently placed in a stereotaxic apparatus (Stoelting, USA), and supplementary doses were given as required. The scalp was dissected, and the skull was cleaned. According to the atlas of Paxinos and Watson (29) for the rat brain, a 30-gauge guide cannula was implanted 1mm above the CA1 area [Antero-posterior (AP)=-3.4 mm; Medio-lateral (ML)=1.5 mm; Dorsal-ventral (DV)=-2.8 mm] for consequent microinjection. Then, a bipolar stimulating electrode was located in the right Schaffer collateral pathway (AP=-4.2 mm; ML=3.8 mm; DV=-2.7−-3.8 mm), and a unipolar recording electrode was taken down from the left side of the skull into the right CA1 zone until the highest response was detected (AP=-3.4 mm; ML=1.5 mm; DV=-4.4−-5.1 mm; at an angle of 52.5 degrees) (29, 30). The electrodes and cannula were slowly lowered (0.2 mm/min) from the cortex to the hippocampus to minimize any trauma to the brain. 

***Electrophysiological recordings***

Extracellular evoked field potentials (fEPSP) were obtained from the CA1 pyramidal cells following stimulation of the Schaffer collateral pathway. Then, it was amplified (×1000) and filtered (band passes 1 Hz to 3 kHz). Signals were passed through an analog-to-digital interface (eLab; Science Beam, Tehran, Iran) to a computer, and data were analyzed using the eProb software (Science Beam; Tehran; Iran). After confirming a stable baseline response, an input-output (I/O) relation was obtained for each animal by regularly varying the stimulus current (100–1000 μA) before LTP induction (30). The stimulus intensity that elicited about 50% of the maximum response was used for all following stimulations. 

Then, the drugs were microinjected with a 2-μl Hamilton syringe into the CA1, in a volume of 0.5 ml, over a 1 min period, 30 min before the inducing of LTP, and was left in place for an extra 60 sec, to facilitate the diffusion. Also, for the interaction group, both antagonists were microinjected simultaneously.

***LTP induction***

After a 30-minute baseline response by applying single pulses of stimulation at 0.1 Hz, LTP was induced by applying an HFS protocol of 100 Hz (4 bursts of 50 stimuli, 0.15 ms stimulus duration, 10 sec inter-burst interval) at an intensity that would elicit approximately 50% of the maximum response (31).

**Figure 1:**
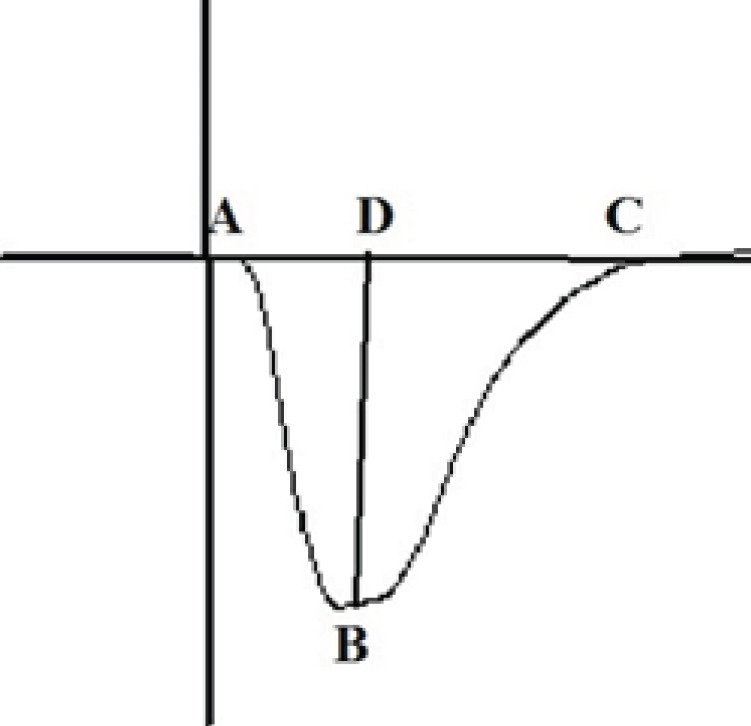
Schematic diagram of fEPSP

 After HFS, baseline stimulation frequency and intensity were resumed. Both field excitatory post-synaptic potential (fEPSP) slope and amplitude were recorded at different times after HFS to determine the changes in the synaptic responses of CA1 neurons. For each time-point, 10 successive evoked responses were averaged at 10 sec stimulus intervals (32). As shown in Figure 1, fEPSP amplitude was calculated as the difference in voltage between the baseline and the negative peak of the fEPSP wave (between B and D), and the fEPSP slope was computed as the slope between the peak of the negative wave and the baseline (between A and B). Changes in the fEPSP amplitude or slope compared to the baseline were plotted in percentages for several time points as a measure of synaptic response.

***Histology***

**Figure 2:**
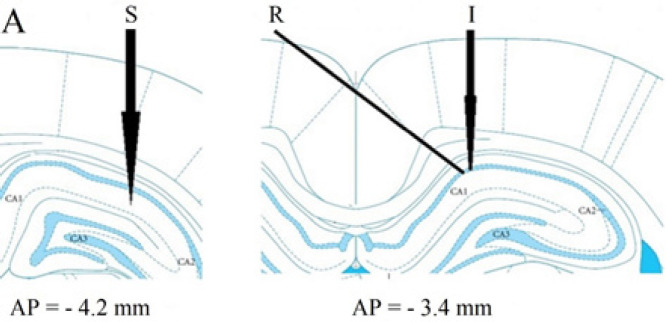
A schematic representation of the Schaffer collateral pathway (left) and CA1 (right) adapted from Paxinos and Watson, showing the site of the stimulating electrode (S) in the Schaffer collateral pathway and recording electrode (R) and injection needle (I) in CA1 region

The shape of field potentials was the main physiological display clue, indicating the electrode’s right location; however, at the end of the experiments, each rat was deeply anesthetized with urethane, then decapitated, and the brain was removed and placed in a formalin solution (10%). After three days, the brain was sliced, and the sites were confirmed according to the atlas of Paxinos and Watson (2007) (29) (Figure 2).

***Statistics***

Data are expressed as mean ± SEM (n = 7). Analysis of data was performed using repeated-measures ANOVA followed by Tukey’s test. The level of statistical significance was set at *P*<0.05.

## Results

***Effect of TCS OX2 29 on the slope and the amplitude of EPSP***

**Figure 3:**
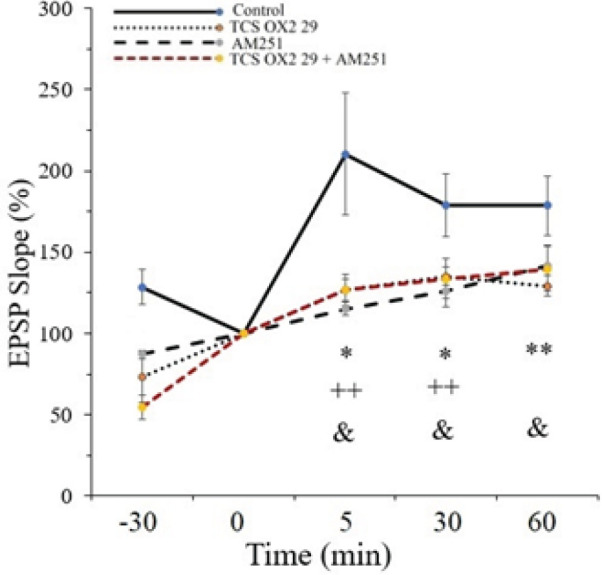
Time-dependent changes in CA1 responses to Schaffer collateral stimulation following an HFS

**Figure 4:**
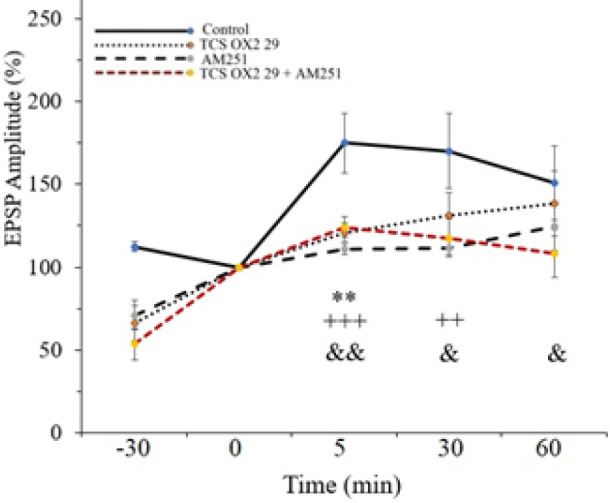
Time-dependent changes in CA1 responses to Schaffer collateral stimulation following an HFS

The effect of HFS on the slope and the amplitude of EPSP in the CA1 area of rat hippocampus were surveyed. The fEPSP slope was intensely increased, resulting in a significant amount of LTP at the Schaffer collateral-CA1 synapses in the control group (210.35±37.42% of pre-HFS baseline) (Figure 3). Our data also shows that the amplitude of fEPSP is 174.9±18.117% of the pre-HFS baseline in the control group (Figure 4).

The results also showed that applying HFS to the Schaffer collateral-CA1 area can increase synaptic transmission in the TCS OX2 29 group (126.56±4.53 % of pre-HFS baseline) (Figure 3). Figure 4 shows that the amplitude of EPSP is 120.93±5.54 % of the pre-HFS baseline in the TCS OX2 29 group. Therefore, the OX2 antagonist significantly decreased fEPSP slope [F (3, 24) =6.028, *P*<0.01] and amplitude [F (3, 24) =3.104, *P*<0.05] in comparison to the control group.

***Effect of AM251 on the slope and the amplitude of EPSP***

Figure 3 displays that after applying HFS to the Schaffer collateral-CA1 area, the EPSP slope was increased to 115.11±4.243 % of the pre-HFS baseline in the AM251 group. Also, Figure 4 shows that the amplitude of EPSP was 110.67±3.24 % of the pre-HFS baseline in the AM251 group. 

Statistical analysis between the AM251 and control groups demonstrated that AM251 causes a significant decrease in the fEPSP slope [F (3, 24) =6.028, *P*<0.001] and amplitude [F (3, 24) =3.104, *P*<0.01] after HFS.

***Effect of co-administration of AM251 and TCS OX2 29 on the slope and amplitude of EPSP***

Figure 3 shows that after applying HFS to the Schaffer collateral-CA1 area, the EPSP slope was increased to 126.75±9.47 % of the pre-HFS baseline in the AM251+TCS OX2 29 group. Figure 4 displays that the amplitude of EPSP was 124.16±6.35 % of the pre-HFS baseline in this group. 

Statistical analysis between the AM251+TCS OX2 29 and control groups shows a significant decrease in the fEPSP slope [F (3, 24) =6.028, *P*<0.01] and amplitude [F (3, 24) =3.104, *P*<0.01] after HFS. It should be said that there was not a significant difference between the group that received both of the antagonists and the groups that received only AM251 into the CA1 region.

**Figure 5:**
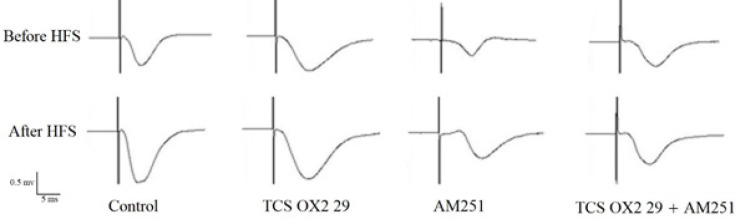
An original trace of the induced field potentials in the CA1 area at the pre-HFS (baseline) and post-HFS time-points

CA1: cornu ammonis1; HFS: high-frequency stimulation

Nevertheless, the inhibitory effect of simultaneous administration of the antagonists on LTP lasted until the end of the recording; as you can see, in Figure 3, there is a significant difference between the AM251+TCS OX2 29 and control groups, as well as between the TCS OX2 29 and control groups, but there is no significant difference between the AM251 and control groups. Figure 4 also shows that there is a significant difference between the AM251+TCS OX2 29 and control groups, while there is no significant difference between groups receiving drugs alone and the control group. Sample traces from each group are demonstrated in Figure 5.

The fEPSP slope was analyzed as AB slope, and the fEPSP amplitude parameters were analyzed as the difference between B and D.

fEPSP: field excitatory post-synaptic potential

CA1: cornu ammonis1

LTP of the EPSP slope in CA1 area synapses of the hippocampus is significantly different between groups. Data are expressed as means ± SEM % of baseline. *: *P*<0.05, **: *P*<0.01 significant difference of TCS OX2 29 compared with control; ++: *P*<0.01 significant difference of AM251 compared with control; &: *P*<0.05 significant difference of TCS OX2 29 + AM251 compared with control.

CA1: cornu ammonis1; HFS: high-frequency stimulation; LTP: long-term potentiation

LTP of the EPSP amplitude in CA1 area synapses of the hippocampus is significantly different between groups. Data are expressed as means ± SEM % of baseline. **: *P*<0.01 significant difference of TCS OX2 29 compared with control; ++: *P*<0.01, +++: *P*<0.001 significant difference of AM251 compared with control; &: *P*<0.05, &&: *P*<0.01 significant difference of TCS OX2 29 + AM251 compared with control.

CA1: cornu ammonis1; HFS: high-frequency stimulation; LTP: long-term potentiation; EPSP: excitatory post-synaptic potential

## Discussion

In this study, we explored the effect of microinjection of the AM251 and TCS OX2 29 into the CA1 area of rat hippocampus on LTP and the functional interaction between CB1R and OX2R of the CA1 in this phenomenon. Our data showed that TCS OX2 29 impairs LTP induction in the Schaffer collateral-CA1 pathway. In accordance with our results, Waynar *et al*. have presented that infusion of orexin A into the DG improves LTP (33). Also**,** Motamedi *et al*. have shown that SB-334867-A (OX1R antagonist) decreases LTP amplification in the DG (17). Furthermore, an experiment on hippocampal slices revealed that orexin A causes the release of GABA, acetylcholine, glutamate, and norepinephrine in the hippocampus; these neurotransmitters facilitate memory and synaptic plasticity (34). 

In the second set of experiments, we found that AM251 impairs LTP induction in the Schaffer collateral-CA1 pathway. As mentioned earlier, there are some controversies around the association of CB1R-mediated signaling in LTP induction. Numerous studies have shown that CB1R antagonists inhibit LTP induction in the CA1 region of the hippocampus (8, 35), while other investigations have revealed that CB1R antagonists do not influence LTP (36, 37). This variation can be a result of some factors containing injection locations, drug doses, model systems, and different stimulation protocols (38). LTP suppression by the AM251 may be mediated by AM251 action on GABAergic interneurons that modulate the glutamatergic neurons (8).

Some studies support the interaction between cannabinoid and orexin systems, reviewed by Berrendero *et al*. (2018) (23) and Flores *et al*. (2013) (39). It has been displayed that OX2R and CB1R can interact with each other in the NAc, but not in the VTA, in the development of the conditioned place preference induced by lateral hypothalamus stimulation (26) and by nicotine (12, 14, 15, 27). In the present study, when CB1R and OX2R antagonists were microinjected concurrently into the CA1, it could significantly inhibit LTP, but not more than when the antagonists were solely microinjected into the nucleus. This suggests that these receptors have a common receptor or post-receptor signaling pathways to apply their effect in the synaptic plasticity process, and there is no synergistic effect between these two antagonists on LTP in the CA1. However, the inhibitory effect of concurrent administration of the antagonists on LTP persisted until the end of the recording. 

## Conclusion

According to our data, although the drugs decreased LTP amplification in the CA1 area, they could not block the induction of LTP. This shows that endogenous cannabinoids and orexins have a modulatory influence on synaptic plasticity through CB1R and OX2R, respectively, in the CA1 region.

Also, there is no interaction between CB1R and OX2R in inducing LTP in the Schaffer collateral-CA1 synapses. This proposes that these receptors presumably act through the same pathway in the area. Nonetheless, other behavioral investigations and molecular techniques are needed to explain this interplay at the signaling level and the pre- or post-receptor cascade involved in controlling this phenomenon.

## Authors’ Contributions

MR N contributed to concept, design, definition of intellectual content, statistical analysis, data analysis, manuscript editing, and manuscript review, and approved the final manuscript. RA T helped with concept, design, manuscript editing, manuscript review, and definition of intellectual content, and approved the final manuscript. R F contributed to design, literature search, experimental studies, data acquisition, manuscript preparation, and statistical analysis, and approved the final manuscript. K G helped conceive and design the study, draft the article and critically revise for important intellectual content, and approved the final manuscript.

## Conflicts of Interest

All authors declare there are no conflicts of interest in this study.
